# Emerging Parasitic Protozoa

**DOI:** 10.3390/pathogens9090704

**Published:** 2020-08-27

**Authors:** Jacob Lorenzo-Morales, José E. Piñero

**Affiliations:** 1 Instituto Universitario de Enfermedades Tropicales y Salud Pública de Canarias, Universidad de La Laguna, Avda. Astrofísico Fco. Sánchez, S/N, La Laguna, Tenerife, 38203 Islas Canarias, Spain; 2 Departamento de Obstetricia, Ginecología, Pediatría, Medicina Preventiva y Salud Pública, Toxicología, Medicina Legal y Forense y Parasitología, Universidad De La Laguna, La Laguna, Tenerife, 38203 Islas Canarias, Spain; 3 Red de Investigación Colaborativa en Enfermedades Tropicales, Instituto de Salud Carlos III, 28029 Madrid, Spain

The terms emerging and re-emerging infectious diseases have been related to a group of diseases that have appeared in a population in the recent past or that have existed but are rapidly increasing in incidence or changing their geographic range. Among the causative agents, parasitic protozoa are emerging as a global health issue worldwide. This special issue is focused on all aspects of parasitic protozoa diseases, from diagnostics to treatment and prevalence, and will have a focalized section on free living amoebae.

Diseases caused by emerging parasitic protozoa present in common the need of standardized diagnostic tools and the lack of fully effective therapeutic agents [1].

In this special issue, supporting the need of therapeutic agents, therapy approaches were described, such as novel natural sources, including leishmanicidal agents. In this work, isolated compounds from a plant called *Withania aristata* were highlighted as new potential agents against these protozoa [2]. Moreover, curcumin derived molecules were presented as useful against *Entamoeba* infections [3], derivates from ursolic acid, which are present in olive leaves among other plants were evaluated as anti-*Acanthamoeba* agents [4], and current approaches to treat *Giardia* spp. infections. The state of the art by scientists working in this field was observed in two of the published papers, one of them dedicated to the development and validation of nanoparticles against *Acanthamoeba* infections [5] and the other highlighting a new powerful group of molecules: statins to treat the lethal encephalitis due to the *Naegleria fowleri* [6]. From a diagnostics point of view, the preliminary results were obtained.

A paper included in this issue showed a high seroprevalence for *Leishmania* in donkeys, as well as the occurrence of parasite DNA in blood [7]. Additional studies would be welcome, both to further investigate the kinetics of antibody response over a longer time and to elucidate the role of the donkey in *Leishmania* epidemiology in endemic areas. 

Altogether, this special issue presents the current work being carried out by experts on the field of emerging parasitic protozoa. As the guest editors of this issue, we would like to thank and congratulate authors involved in it and also to encourage their research in this field.
